# Protective Efficacy of N-(2-Hydroxyphenyl) Acetamide against Adjuvant-Induced Arthritis in Rats

**DOI:** 10.1155/2013/635143

**Published:** 2013-07-18

**Authors:** Kahkashan Perveen, Farina Hanif, Huma Jawed, Shabana U. Simjee

**Affiliations:** ^1^Dr. Panjwani Center for Molecular Medicine and Drug Research, International Center of Chemical and Biological Sciences, University of Karachi, Karachi 75270, Pakistan; ^2^H.E.J. Research Institute of Chemistry, International Center of Chemical and Biological Sciences, University of Karachi, Karachi 75270, Pakistan

## Abstract

Rheumatoid arthritis is a chronic inflammatory joint disease characterized by synovial proliferation and tissue destruction. Proinflammatory cytokines like interleukin-1beta (IL-1**β**) and tumor necrosis factor-alpha (TNF-**α**) play a key role in the disease process and elevate energy expenditure, which further increases the joint pain and stiffness. To explore the effects of N-(2-hydroxyphenyl) acetamide (NA-2) on the development of arthritis, clinical signs, histopathology of knee joints, nociception analysis, and the serum levels of IL-1**β** and TNF-**α** were monitored. Arthritis was induced by intradermal administration of heat-killed adjuvant *Mycobacterium tuberculosis* H37Ra in rats. NA-2 and indomethacin treatments were started in their respective group on the same day when adjuvant was administered. Experiments were terminated when arthritic score of 4 was observed in arthritic control group. NA-2 (5 mg/kg) treatment significantly ameliorated the disease severity. Reduction in body weight and increase in paw oedema were significantly reversed in arthritic animal receiving NA-2. The nociceptive sensation was also inhibited in the NA-2 treated arthritic rats. Remission was associated with improved histology and significant decreased expression of serum proinflammatory cytokines (*P* < 0.05 for IL-1**β** and TNF-**α**). Based on our observations, it can be suggested that NA-2 possesses promising anti-arthritic property, and it can be used as a therapeutic agent for arthritis.

## 1. Introduction

Rheumatoid arthritis (RA) is a destructive inflammatory polyarticular joint disease characterized by a severe progressive synovitis in peripheral joints followed by destruction of joints and ankylosis [1, 2]. Histopathological characterization includes immense synovial proliferation and subintimal infiltration of inflammatory cells, which along with angiogenesis leads to the formation of an aggressive tissue called pannus [3, 4]. Further growth of pannus causes bone erosion and cartilage thinning which result in loss of joint function. This pannus is considered as local tumor in early stage of disease, and most of the nutrient and oxygen of the body are delivered towards this proliferating pannus [5]. Although the exact etiology of this autoimmune joint disease is still unknown, it is believed that primary cause may include constitutive activation of immune cells [6] which may result in increasing production of proinflammatory cytokines and abnormal sensing of self-antigen as nonself due to their similarity with a foreign protein [7–12]. These cytokines are also responsible for the various symptoms related to the disease including a drastic drop in the body weight and cachexia both in animal models and human due to hypermetabolism [13–15].

The most commonly used treatment to manage the outcome and progression of RA includes nonsteroidal anti-inflammatory drugs, disease modifying antirheumatic drugs, and glucocorticoids [16–20]. The prolongs use of these drugs cause moderate to severe side effects and only controls the symptoms of the disease but does not improve the quality of life. Therefore the goal of the present study is to evaluate the therapeutic activity of N-(2-hydroxyphenyl) acetamide (NA-2). NA-2 is a derivative of salicylic acid and has shown very promising anti-inflammatory activity in our pilot experiments. Therefore we aimed to explore its antiarthritic activity in adjuvant-induced arthritis model in rats.

## 2. Materials and Methods

### 2.1. Animals

Female Sprague Dawley rats were divided into five groups with 12 animals in each group. Animals were kept in temperature and humidity controlled environment with a 12/12 h light and dark cycle and free access to laboratory food pallet and water. Ethical guidelines of the International Association for the Study of Pain [21, 22] in conscious animals and the guidelines set by the Scientific Advisory Committee on Animal Care, and Use at International Center for Chemical and Biological Sciences, University of Karachi (Protocol no. 1209004), for the animal handling were applied during entire study. 

### 2.2. Induction of Arthritis

Arthritis was induced by a single intradermal injection of freshly prepared suspension (1 mg/0.1 mL) of lyophilize *Mycobacterium tuberculosis* MT37Ra (Difco Laboratories, USA). Adjuvant was injected intradermally at the tail base under anesthesia and the animals were monitored closely until they regain consciousness. Treatment was started on the same day after induction of arthritis.

### 2.3. Treatment

Indomethacin (5 mg/kg) and the test drug NA-2 (5 mg/kg) were daily administered i.p. starting from day of arthritis induction, that is, the day when adjuvant was administered until the end of experiment. The dose of the test drug was selected by preliminary dose finding studies in our laboratory.

### 2.4. Assessment of Arthritis

Rats were accessed for arthritic index on alternate days from 0–4 point scale (Table 1). The severity of diseases was calculated by adding the scores of each individual paw which were maximum 16 for each animal. The scoring system used in the study is shown in Table 1. Severity of arthritis was also measured by quantitative change in the body weights, paw volume oedema, and pain threshold on alternate days.

### 2.5. Histology of Knee Joint

On the day when experiment was terminated, animals were humanely sacrificed, and the knee joints of each animal were collected and processed for histological evaluation. The joints were decalcified, fixed, processed, embedded, cut, and stained with haematoxylin and eosin. These processed sections were then observed under light microscope (Olympus BX41), and images were captured by Olympus DP 12 camera and visualized using Olysia software.

### 2.6. Measurement of Proinflammatory Cytokines Interleukin-1beta (IL-1*β*) and Tumor Necrosis Factor-Alpha (TNF-*α*) by ELISA

The quantitative measurement of IL-1*β* and TNF-*α* was performed in the serum samples collected from the normal, arthritic, and treated groups using ELISA kits (Thermo Fisher Scientific Inc., USA, for TNF-*α* and CUSABIO BIOTECH, China, for IL-1*β*). Each sample or standard was run in duplicates, and the data were then averaged.

### 2.7. Statistical Analysis

Data were reported as mean ± SEM. The statistical analysis was performed using statistical package for the social sciences (SPSS 19) software. One-way analysis of variance (ANOVA) was used to analyze the data. The Bonferroni's post hoc test was used to determine mean difference between the groups.

## 3. Results

### 3.1. Effect of NA-2 on Body Weight in Adjuvant-Induced Arthritic Rats

Animals in various groups were weighed throughout the experiment starting from day 0 until the end of the experiment (day 22).  Table 2 demonstrates the change in the body weights of the control and test groups during the study period. In comparison to the normal control group, body weights of arthritic rats were significantly reduced from day 14 (*P* < 0.038) till the end of 22-day experiment (*P* < 0.003). However, compared to the arthritic control group, ANOVA performed on the data showed that both treatments, that is, indomethacin and NA-2 treatments significantly prevented the body weight loss (*P* < 0.05).

### 3.2. Effect of NA-2 on Paw Oedema in Adjuvant-Induced Arthritic Rats

Table 3 shows the effect of NA-2 on paw oedema in treated and untreated arthritic animals. It was observed that the paw oedema of arthritic control rats was markedly increased from day 4. The statistical analysis revealed that the increase in the paw oedema of the arthritic control group was significantly higher than the normal control group on day 14 (*P* < 0.05) onward. In contrast, the NA-2 (*P* < 0.05) and indomethacin (*P* < 0.002) treatments significantly inhibited the increase in the paw volume.

### 3.3. Effect of NA-2 on Pain Threshold in Adjuvant-Induced Arthritic Rats

**Figure 1:**
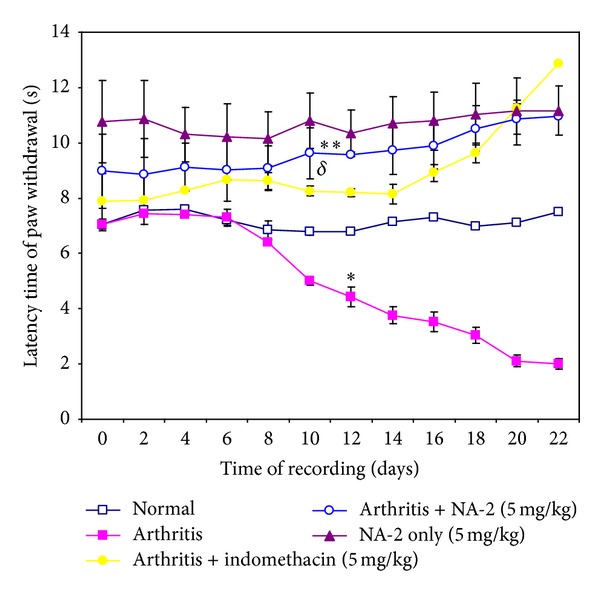
Effect of NA-2 (5 mg/kg) on the latency of paw withdrawal from the thermal stimulus induced by radiant heat in arthritic and nonarthritic rats. Each value in line graph represents Mean ± SEM of 12 animals/group. A significant decrease (*P < 0.0027) in the latency time of arthritic animals compared to normal animals was observed from day 12 onward. Treatment with NA-2 given 30 minutes before the test was conducted exhibiting an increase in latency time of animals from day 8 which significantly increases (^*δ*^ *P* < 0.05) from day 12 as compared to nontreated arthritic group. Treatment with indomethacin also increases latency time from day 8 which becomes significant (**P < 0.05) from day 12 compared to nontreated arthritic group till the end of experiment.

Effect of NA-2 on nociception of planter region with progression of the disease is shown in Figure 1. In case of arthritic control group, latency time gradually decreases throughout the experiment, and it was significantly lower than the normal control on day 12 onward (*P* < 0.05). The indomethacin or NA-2 treatment exhibited a pronounced antinociceptive effect in arthritic treated group. Within the treatment groups, the NA-2 treated group was found to exhibit slightly better activity from day 8 in comparison to the indomethacin treated group; however, the significant difference was not obvious until day 12. ANOVA revealed that the activity was significantly higher than arthritic control group over a period of 22 days (*P* < 0.05).

### 3.4. Effect of NA-2 on Histopathology of Knee Joints

The numerical value of histological examinations is shown in Table 4. There was an increase in the inflammatory score and bone erosion in arthritic control samples compared to the normal group. There was also a prominent inhibition of both infiltration of inflammatory cells and bone erosion after the treatment with NA-2.

### 3.5. Effect of NA-2 on Serum Proinflammatory Cytokines (IL-1*β* and TNF-*α*)

**Figure 2:**
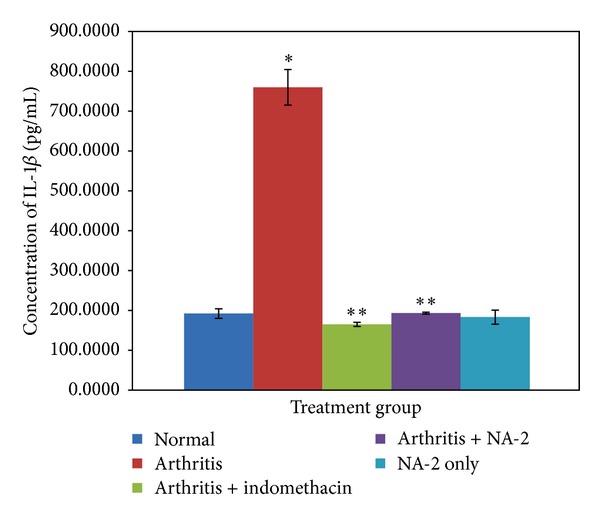
Effect of NA-2 on serum levels of IL-1*β* in arthritic and nonarthritic animals. A significant increase (*P < 0.05) in the levels of serum IL-1*β* was found in the arthritic control group compared to normal animals. In contrast to the arthritic control group, both the indomethacin and NA-2 treatments were observed to decrease the IL-1*β* in the serum of the treated animals (**P < 0.05) which was comparable to that of the normal control group.

**Figure 3:**
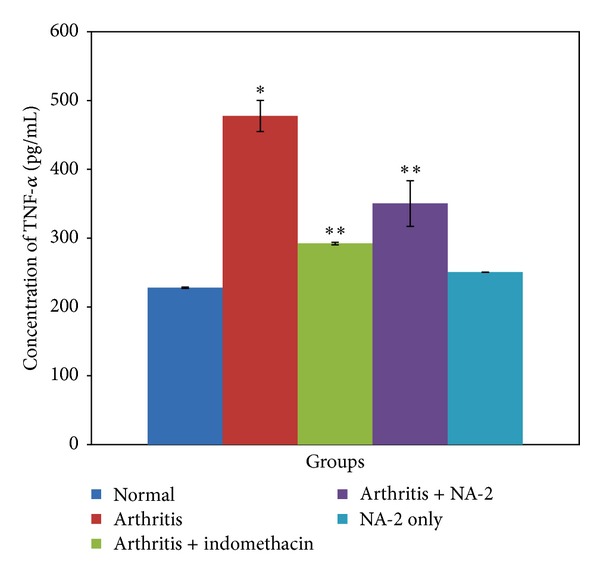
Serum TNF-*α* measured in the arthritic and nonarthritic samples. In comparison to the normal control group, the arthritic control animals demonstrated a marked increase in their serum levels of TNF-*α* (*P < 0.05). In contrast to the arthritic control group, the measured cytokine levels were observed to be significantly reduced following the treatment with NA-2 or indomethacin (**P < 0.05). Within the treatment groups, no significant difference was found.

The levels of IL-1*β* and TNF-*α* in the serum samples collected at the end of 22 days were determined by ELISA (Figures 2 and 3). Arthritic control group showed significant increase in the levels of IL-1*β* and TNF-*α* (*P* < 0.05) when compared with the normal control group. The animals treated with indomethacin or NA-2 exhibited a significant decrease in the levels of both these cytokines (*P* < 0.05) in comparison to the arthritic control. When the treatment groups were compared with each other, the ANOVA with Bonferroni's test revealed no significant difference between indomethacin and NA-2 treated groups.

## 4. Discussion

Adjuvant-induced arthritis (AIA) in rodents is a widely used model for testing and developing antiarthritic agents. In the present study, we have used SD rats which are considered to be a moderate responder strain to AIA and demonstrated that, following intradermal administration of MT37Ra suspension, the SD rats developed full blown arthritis at 100% incidence. Since our results were readily reproducible and validity of the model was proved, therefore we have used it to study the effect of NA-2 on the development process of the arthritic disease. 

Adjuvant-induced arthritis is characterized by both short- and long-term inflammatory changes and associated secondary thermal hyperalgesia [23–25] which persist until the end of experiment. The appearance of thermal hyperalgesia at the paw in the course of adjuvant-induced arthritis can be interpreted as secondary hyperalgesia. Hypersensitivity to noxious stimuli in inflamed regions is a well-known phenomenon in experimental animals and humans [26, 27]. It is reported that the hyperalgesia is produced by central sensitization due to hyperexcitability of spinal cord neurons during peripheral pathologic process [28]. During the development of inflammation in the joints in arthritic condition, the spinal neurons become hyperresponsive to the stimuli [29]. These studies supported our observation made in case of arthritic control group. However, the systemic administration of NA-2 significantly reduced thermal hyperalgesia of the paw on day 8 onward thereby indicating that secondary hyperalgesia is also attenuated. The significant reduction of these symptoms with NA-2 clearly shows that it has strong potential to decrease inflammation evoked nociception. The antinociceptive effect of NA-2 was observed along with the marked reduction in the inflammation as it was obvious from the reduction of paw oedema. The histological analysis of the knee joints also revealed massive changes in the infiltrating inflammatory cells and tissue erosion as seen in the case of arthritic control group. In comparison to the arthritic control group, the NA-2 treated animals exhibited significantly attenuated inflammation-induced changes in joints and in pain threshold. The NA-2 induced reduction in the responses of the animals to the thermal algesia shows that it has a potent antinociceptive effect under inflammatory conditions in AIA model. 

Number of studies has suggested that macrophages, proliferating synovial cells, and T lymphocytes play a major role in the pathogenesis of RA disease [30–34]. The pro-inflammatory cytokines such as IL-1*β* and TNF-*α*, produced by the activated macrophages and T cells, appear to be involved in the perpetuation of arthritis. Increased levels of these cytokines have been extensively reported in the synovial fluid of both the RA patients and arthritic animals [35–39] and are therefore considered to play an important role in the pathogenesis of arthritis. The regulation of these cytokine levels in arthritic subjects is considered as one of the approaches to the treatment of arthritis. Therefore, we also included these parameters to monitor the effect of the NA-2 treatment in the arthritic model. 

In correlation to previous studies, we have observed a dramatic increase in serum levels of IL-1*β* and TNF-*α* in relation to the disease progression. However, the treatment of NA-2 actually demonstrated an overall protective effect as an anti-inflammatory which produces a significant inhibition of IL-1*β* and TNF-*α*. The TNF-*α* promotes the release of IL-1*β* which in turn upregulates the expression of cycloxygenase-2 (COX-2) [40]. Prostaglandins formed by COX-2 then further aggravate the inflammation and hyperalgesia in AIA [41–43]. It is probable that NA-2 exerts its analgesic effect on AIA by inhibiting IL-1*β* and TNF-*α* production and consequently decreasing COX-2 expression triggered by the cytokine. The histological analysis of the knee joints collected from the NA-2 treated arthritic group also revealed a positive correlation of the inhibitory effect of the treatment on IL-1*β* and TNF*α* with the ability of NA-2 to prevent cartilage and bone erosion. 

## 5. Conclusion

Thus in the present study, we have demonstrated that NA-2 prominently suppresses inflammation and inhibits the development of arthritis in AIA model of rats. Furthermore, we have also concluded that a compound having dual action on both the proinflammatory cytokines and hyperalgesia may be an approach to reduce the tissue damage and inflammation associated with rheumatic arthritis. In summary, the results of the present study strongly suggest that NA-2 treatment can reduce hyperalgesic states probably by downregulating IL-1*β* and TNF-*α*, even in the presence of mild inflammation in an animal model of RA. It demonstrated a profound effect on the inflammatory joint pain. In general, the efficacy of this compound as antirheumatic drugs has been evaluated based on application in chronic treatment. As demonstrated in AIA, analgesic effects may be found before the onset of antirheumatic or anti-inflammatory effects in our model. 

## Figures and Tables

**Table 1:** Macroscopic arthritic scoring system used for the clinical scoring of the induced arthritis.

Arthritic score	Observations
0	No signs of arthritis
1	Swelling and/or redness of the paw or one digit
2	Two joints involved
3	More than two joints involved
4	All joints were involved with severe arthritis of entire paw and digits

The score was based on the number of joints involved, the severity, and extent of the erythema and edema of the tissue.

**Table 2:** Effect of NA-2 (5 mg/kg dose) on the time course of the development of disease reflected by decrease in body weight over a period of 22 days.

Experimental group	Body weight (in gms)/day of recording											
	00	02	04	06	08	10	12	14	16	18	20	22
Normal	193.0 ± 4.5	193.4 ± 4.2	193.8 ± 6.2	195.7 ± 5.9	197.6 ± 5.6	200 ± 5.8	200.5 ± 5.9	201.6 ± 5.9	202.5 ± 5.9	203.2 ± 6.1	204 ± 5.8	204.1 ± 5.9
Arthritic control	192.9 ± 3.8	192.9 ± 3.8	193.8 ± 6.2	191.8 ± 4.1	191.1 ± 3.8	190.4 ± 3.5	188.81 ± 3.2*	187 ± 3.0	185.2 ± 3.1	182.5 ± 6.0	180.2 ± 5.9	179.5 ± 6.1
Arthritis + indomethacin (5 mg/kg)	194 ± 7.4	194 ± 7.3	194.1 ± 7.1	196.5 ± 7.6	196.6 ± 6.5	196.6 ± 7.5	196.6 ± 7.5**	198.2 ± 7.1	199.7 ± 7.7	200 ± 7.5	200.7 ± 7.7	199.5 ± 6.9
Arthritis + NA-2 (5 mg/kg)	188.5 ± 7.4	188.6 ± 5.1	188.7 ± 4.3	188.7 ± 3.9	188.9 ± 3.6	188.3 ± 4.4	187.9 ± 5.0	186.8 ± 4.7	185.7 ± 4.5	186.7 ± 4.7^#^	187.1 ± 5.0	187.6 ± 6.1
NA-2 only (5 mg/kg)	193.5 ± 8.4	192.6 ± 8.4	192.3 ± 8.2	192.8 ± 8.3	193.3 ± 8.3	195.0 ± 8.0	196.3 ± 7.4	197.8 ± 7.5	200.6 ± 7.2	201.5 ± 7.4	202.44 ± 7.1	202.6 ± 7.4

The value in the table represents the mean ± SEM of 12 animals/group. A significant decrease in body weights from day 12 (*P< 0.038) of arthritic animal compared to normal animals was observed till the end of experiment. Treatment with indomethacin significantly (**P < 0.05) increases body weights compared to nontreated arthritic group from day 12 till the end of experiment. Treatment with NA-2 increases body weight of animals, but it shows significant increase (^#^ *P* < 0.05) from day 18 compared to arthritic group.

**Table 3:** Effect of NA-2 (5 mg/kg dose) on the time course of the development of inflammation as shown by increase in paw volume.

Experimental group	Mean ± SEM of paw volume (mL)/day of recording											
	00	02	04	06	08	10	12	14	16	18	20	22
Normal	3.9 ± 0.5	3.9 ± 0	3.9 ± 0.72	3.8 ± 0.72	3.7 ± 0.67	3.8 ± 0.75	3.8 ± 0.73	4.0 ± 0.74	4.0 ± 0.63	4.0 ± 0.61	4.0 ± 0.51	4.0 ± 0.53
Arthritic control	4.4 ± 0.39	4.4 ± 0.39	4.1 ± 0.39	4.0 ± 0.59	4.1 ± 0.39	4.3 ± 0.67	4.5 ± 0.53	4.8 ± 0.46	4.9 ± 0.00	5.3 ± 0.70	5.4 ± 0.70	5.7 ± 0.84
Arthritis + indomethacin (5 mg/kg)	4.1 ± 0.54	4.1 ± 0.52	4.0 ± 0.67	4.0 ± 0.66	4.0 ± 0.67	4.1 ± 0.00	3.9 ± 0.0^##^	3.8 ± 0.00	4.0 ± 0.00	4.0 ± 0.00	3.9 ± 0.00	3.8 ± 0.00
Arthritis + NA-2 (5 mg/kg)	4.1 ± 0.59	4.0 ± 0.68	3.9 ± 0.72	4.0 ± 0.66	4.0 ± 0.85	4.0 ± 0.00	3.9 ± 0.61^#^	4.1 ± 0.50	4.3 ± 0.60	4.3 ± 0.58	4.3 ± 0.59	4.3 ± 0.57
NA-2 only (5 mg/kg)	4.2 ± 0.64	3.9 ± 0.85	3.9 ± 0.75	4.1 ± 0.75	4.1 ± 0.78	3.8 ± 0.93	4.0 ± 0.00	4.0 ± 0.00	4.0 ± 0.63	4.0 ± 0.62	4.0 ± 0.64	4.0 ± 0.62

The value in the table represents the mean ± SEM of 12 animals/group. A significant increase in paw volume (*P* < 0.034) of arthritic animal compared to normal animals was observed from day 12 onward (*P < 0.00) until the end of experiment. Treatment with indomethacin decreases paw volume significantly (^##^ *P* < 0.05) from day 12 till the end. Treatment with NA-2 decreases paw volume of arthritic which significantly decreases from day 12 (^#^ *P* < 0.00) compared to control arthritic group.

**Table 4:** Histological scores for arthritic and nonarthritic rats.

Group	Knee joint	
	Inflammation	Erosion
Normal	0	0
Arthritis	4	4
Arthritis + indomethacin (5 mg/kg)	3	2
Arthritis + NA-2 (5 mg/kg)	2	0

The severity of arthritis was scored on a scale of 0–4. A score of 0 indicates absence of inflammation (I) or erosion (E) in the joints and tail samples, whereas a score of 2-3 demonstrates a mild to moderate inflammation and tissue erosion.

## References

[1] Harris ED (1990). Rheumatoid arthritis. Pathophysiology and implications for therapy. *The New England Journal of Medicine*.

[2] Feldmann M, Brennan FM, Maini RN (1996). Role of cytokines in rheumatoid arthritis. *Annual Review of Immunology*.

[3] Murunikkara V, Pragasam SJ, Kodandaraman G, Sabina EP, Rasool M (2012). Anti-inflammatory effect of piperine in adjuvant-induced arthritic rats: a biochemical approach. *Inflammation*.

[4] Koch AE (1998). Angiogenesis: implications for rheumatoid arthritis. *Arthritis & Rheumatism*.

[5] Folkman J (1995). Angiogenesis in cancer, vascular, rheumatoid and other disease. *Nature Medicine*.

[6] Feige U, Hu Y-L, Gasser J, Campagnuolo G, Munyakazi L, Bolon B (2000). Anti-interleukin-1 and anti-tumor necrosis factor-*α* synergistically inhibit adjuvant arthritis in lewis rats. *Cellular and Molecular Life Sciences*.

[7] Van den Berg WB (2001). Uncoupling of inflammatory and destructive mechanisms in arthritis. *Seminars in Arthritis and Rheumatism*.

[8] Rioja I, Bush KA, Buckton JB, Dickson MC, Life PF (2004). Joint cytokine quantification in two rodent arthritis models: kinetics of expression, correlation of mRNA and protein levels and response to prednisolone treatment. *Clinical and Experimental Immunology*.

[9] Campagnuolo G, Bolon B, Feige U (2002). Kinetics of bone protection by recombinant osteoprotegerin therapy in Lewis rats with adjuvant arthritis. *Arthritis and Rheumatism*.

[10] Gravallese EM (2002). Bone destruction in arthritis. *Annals of the Rheumatic Diseases*.

[11] Stolina M, Adamu S, Ominsky M (2005). RANKL is a marker and mediator of local and systemic bone loss in two rat models of inflammatory arthritis. *Journal of Bone and Mineral Research*.

[12] Hazenberg MP, Klasen IS, Kool J, Ruseler-Van Embden JGH, Severijnen AJ (1992). Are intestinal bacteria involved in the etiology of rheumatoid arthritis?. *APMIS*.

[13] Roubenoff R, Roubenoff RA, Cannon JG (1994). Rheumatoid cachexia: cytokine-driven hypermetabolism accompanying reduced body cell mass in chronic inflammation. *Journal of Clinical Investigation*.

[14] Roubenoff R, Freeman LM, Smith DE, Abad LW, Dinarello CA, Kehayias JJ (1997). Adjuvant arthritis as a model of inflammatory cachexia. *Arthritis and Rheumatism*.

[15] Rall LC, Roubenoff R (1996). Body composition, metabolism, and resistance exercise in patients with rheumatoid arthritis. *Arthritis Care and Research*.

[16] Choy EHS, Panayi GS (2001). Cytokine pathways and joint inflamation in rheumatoid arthritis. *The New England Journal of Medicine*.

[17] O’Dell JR (2004). Therapeutic strategies for rheumatoid arthritis. *The New England Journal of Medicine*.

[18] Firestein GS, Harris ED, Budd RC, Genovese MC, Firestein GS, Sargent JS, Sledge CB (2005). Etiology and pathogenesis of rheumatoid arthritis. *Kelley's Textbook of Rheumatology*.

[19] McDuffie FC (1985). Morbidity impact of rheumatoid arthritis on society. *American Journal of Medicine*.

[20] Smolen JS, Steiner G (2003). Therapeutic strategies for rheumatoid arthritis. *Nature Reviews Drug Discovery*.

[21] Zimmermann M (1983). Ethical guidelines for investigations of experimental pain in conscious animals. *Pain*.

[22] Bakharevski O, Stein-Oakley AN, Thomson NM, Ryan PFJ (1998). Collagen induced arthritis in rats. Contrasting effect of subcutaneous versus intradermal inoculation of type II collagen. *Journal of Rheumatology*.

[23] Colpaert FC (1987). Evidence that adjuvant arthritis in the rat is associated with chronic pain. *Pain*.

[24] Nanayama T, Kuraishi Y, Ohno H, Satoh M (1989). Capsaicin-induced release of calcitonin gene-related peptide from dorsal horn slices is enhanced in adjuvant arthritic rats. *Neuroscience Research*.

[25] Honoré P, Menning PM, Rogers SD (1999). Spinal substance P receptor expression and internalization in acute, short-term, and long-term inflammatory pain states. *Journal of Neuroscience*.

[26] Ren K, Dubner R (1999). Inflammatory models of pain and hyperalgesia. *ILAR Journal*.

[27] Schaible H-G, Schmelz M, Tegeder I (2006). Pathophysiology and treatment of pain in joint disease. *Advanced Drug Delivery Reviews*.

[28] Bajaj P, Bajaj P, Graven-Nielsen T, Arendt-Nielsen L (2001). Osteoarthritis and its association with muscle hyperalgesia: an experimental controlled study. *Pain*.

[29] Woolf CJ, Salter MW (2000). Neuronal plasticity: increasing the gain in pain. *Science*.

[30] Schaible H-G, Grubb BD (1993). Afferent and spinal mechanisms of joint pain. *Pain*.

[31] Kinne RW, Bräuer R, Stuhlmüller B, Palombo-Kinne E, Burmester G-R (2000). Macrophages in rheumatoid arthritis. *Arthritis Research*.

[32] Keffer J, Probert L, Cazlaris H (1991). Transgenic mice expressing human tumour necrosis factor: a predictive genetic model of arthritis. *EMBO Journal*.

[33] Bingham CO (2002). The pathogenesis of rheumatoid arthritis: pivotal cytokines involved in bone degradation and imflammation. *Journal of Rheumatology*.

[34] Rommel C, Camps M, Ji H (2007). PI3K*δ* and PI3K*γ*: partners in crime in inflammation in rheumatoid arthritis and beyond?. *Nature Reviews Immunology*.

[35] Huber LC, Distler O, Tarner I, Gay RE, Gay S, Pap T (2006). Synovial fibroblasts: key players in rheumatoid arthritis. *Rheumatology*.

[36] Panayi GS, Lanchbury JS, Kingsley GH (1992). The importance of the T cell in initiating and maintaining the chronic synovitis of rheumatoid arthritis. *Arthritis and Rheumatism*.

[37] Szekanecz Z, Koch AE, Kunkel SL, Strieter RM (1998). Cytokines in rheumatoid arthritis. Potential targets for pharmacological intervention. *Drugs and Aging*.

[38] Gravallese EM, Goldring SR (2000). Cellular mechanism and the role of cytokines in bone erosions in rheumatoid arthritis. *Arthritis & Rheumatism*.

[39] Fan AY, Lao L, Zhang RX (2005). Effects of an acetone extract of *Boswellia carterii* Birdw. (Burseraceae) gum resin on adjuvant-induced arthritis in lewis rats. *Journal of Ethnopharmacology*.

[40] Cai X, Zhou H, Wong YF (2007). Suppression of the onset and progression of collagen-induced arthritis in rats by QFGJS, a preparation from an anti-arthritic Chinese herbal formula. *Journal of Ethnopharmacology*.

[41] Crofford LJ, Wilder RL, Ristimaki AP (1994). Cyclooxygenase-1 and -2 expression in rheumatoid synovial tissues. Effects of interleukin-1*β*, phorbol ester, and corticosteroids. *Journal of Clinical Investigation*.

[42] Anderson GD, Hauser SD, McGarity KL, Bremer ME, Isakson PC, Gregory SA (1996). Selective inhibition of cyclooxygenase (COX)-2 reverses inflammation and expression of COX-2 and interleukin 6 in rat adjuvant arthritis. *Journal of Clinical Investigation*.

[43] Portanova JP, Zhang Y, Anderson GD (1996). Selective neutralization of prostaglandin E2 blocks inflammation, hyperalgesia, and interleukin 6 production in vivo. *Journal of Experimental Medicine*.

